# Recurrent Hypothermia and Autonomic Dysfunction Secondary to Shapiro Syndrome

**DOI:** 10.1002/acn3.70419

**Published:** 2026-04-29

**Authors:** Naveen Kumar, Jeremy Johnson, Susanne Watkins, Eoin Mulroy

**Affiliations:** ^1^ Department of Neurology King's College Hospital London UK; ^2^ Institute of Psychiatry, Psychology & Neuroscience King's College London London UK; ^3^ Faculty of Life Sciences and Medicine King's College London London UK

**Keywords:** autonomic dysfunction, hypothermia, shapiro syndrome, thermoregulation

## Abstract

A 44‐year‐old man presented with recurrent hypothermia, diaphoresis and hypertension. Extensive investigation for infectious, inflammatory, metabolic and endocrine aetiologies was negative. MR scan of the brain demonstrated no lesions but revealed callosal dysgenesis, consistent with Shapiro syndrome. We describe the pathophysiologic basis of central thermoregulation and an approach to the assessment and management of patients presenting with disorders thereof, including the importance of considering central thermoregulatory failure after systemic disease has been carefully excluded.

## 1 Case Description

A 44‐year‐old man presented to hospital complaining of feeling cold, sweaty, weak, and confused. He had been well in preceding days and had no infective symptoms. His most recent foreign travel had been one year earlier, to Sierra Leone. His medical history was significant for hypertension treated with amlodipine 10 mg daily.

On examination, he was restless and diaphoretic. His blood pressure was 207/176 mmHg and his temperature was 33.5°C. He had a normal mental state other than transient auditory hallucinations at the time of initial arrival. The neurological examination was normal. Blood results at presentation are summarised in Table S1.

On the presumption of an infectious aetiology, he received empiric intravenous acyclovir and co‐amoxiclav and oral antihypertensives. His condition improved with antimicrobial treatment and active external rewarming over 48 h. Further workup for endocrinopathy, autoimmune and infectious processes, and systemic malignancy did not show any abnormalities (summarised in Table S2). CT of the chest, abdomen and pelvis and whole‐body CT PET were normal. MR scan of the brain was reported as normal. He experienced several more episodes of transient hypothermia associated with hypertension and sweating during his four‐week admission, all of which resolved spontaneously.

Over the following 4 years, the patient was admitted over a dozen times with identical symptoms, that is, hypothermia as low as 28°C, alongside diaphoresis, blood pressure disturbance (predominantly hyper‐, but also profound hypotension) and symptomatic hyponatremia between 119 and 132 mmol/L. Repeat testing on each occasion showed no evidence of infection or endocrinopathy. These repeated presentations with periodic hypothermia, autonomic disturbance and hyponatraemia led us to consider a thermoregulatory disorder of central origin.

**FIGURE 1:**
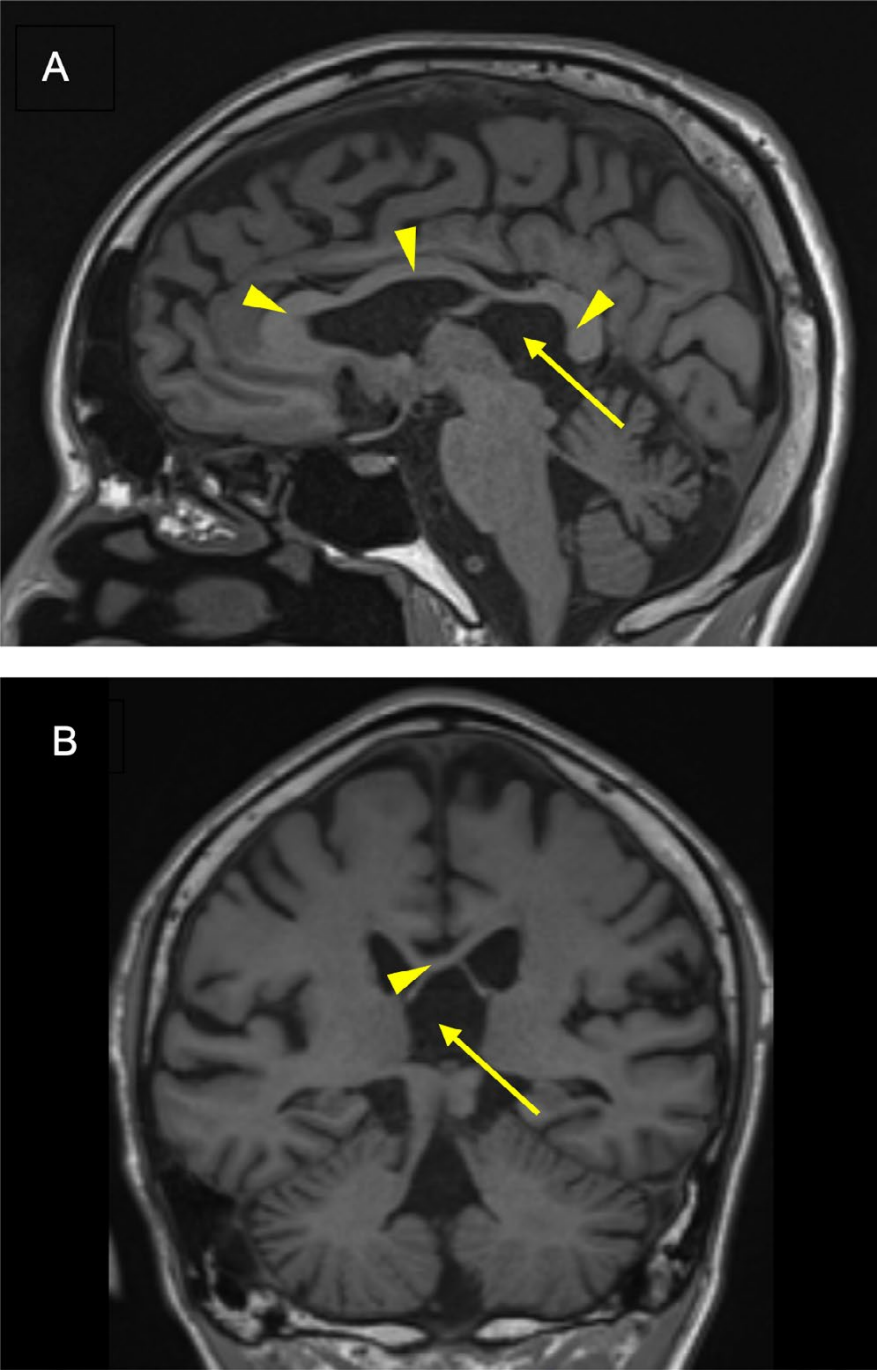
MR scan of brain. (A) Sagittal T2‐FLAIR‐weighted image; (B) Coronal T2‐FLAIR‐weighted image demonstrating dysgenesis of the corpus callosum (yellow arrowheads) and cavum veli interpositi (yellow arrow).

Review of prior imaging revealed a previously overlooked abnormality of the corpus callosum, together with a cavum veli interpositi (Figure 1), supportive of Shapiro syndrome.

## 2 Discussion

### 2.1 How and Why Did We Evolve to Control Body Temperature?

The evolution of mammals from sea‐ to land‐based existence challenged thermoregulatory capabilities, given the higher variability of land temperatures compared to aquatic environments. This drove a transition from poikilothermy (variable body temperature, depending on environmental conditions) to homeothermy (the ability to control internal temperature within narrow set ranges, maximising the benefits of high‐temperature existence such as increased enzymatic activity, faster neural processing, and better healing) [1]. For mammals, the development of endothermy (the ability to control temperature via endogenous heat production) offered a survival advantage when competing against ectothermic dinosaurs, who relied on environmental sources of heat such as sunlight to maintain their temperature, and hence were inactive at night, more susceptible to the effects of seasonal variation, and in some cases limited to certain latitudes [2].

**FIGURE 2:**
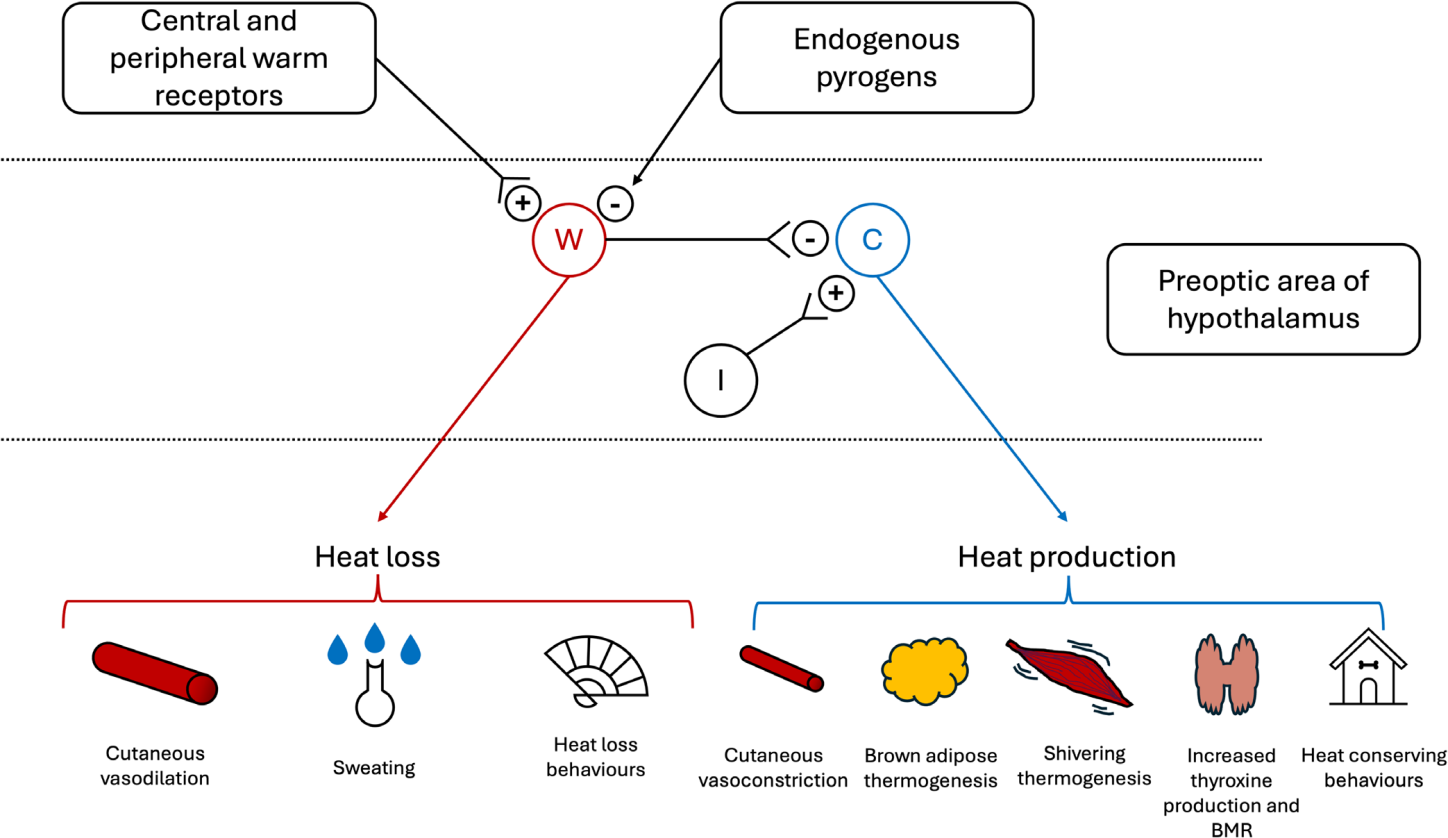
Input and output of three different populations of neurons in the pre‐optic area (POA) of the anterior hypothalamus. Warm sensing neurons (W) show increased activity in response to warm and receive additional excitatory input from afferents carrying information from central and peripheral warm thermoreceptors. Increased activity in W neurons leads to activation of effector mechanisms to drive heat loss, including sweating and peripheral vasodilation. Cold sensing neurons (C) make up less than 5% of the total population of the POA and receive tonic inhibitory input from W neurons, which is balanced against tonic excitatory input from temperature‐insensitive neurons (I). Disinhibition of C neurons leads to activation of effector mechanisms to produce and maintain heat, including thermogenesis (shivering and non‐shivering) and peripheral vasoconstriction. The production of endogenous pyrogens secondary to infection or inflammation leads to reduced activity in W neurons, suppressing the warm response and raising the temperature, as seen in fever. BMR, basal metabolic rate.

In humans, body temperature is stringently controlled, with core temperature generally maintained in the range 36°C to 37.5°C^3^. This temperature optimises the balance between efficient metabolic processing and avoiding the damaging effects of excess heat. Thermoregulatory responses, orchestrated by a hypothalamic ‘thermostat’, aim to maintain core body temperature within these strict parameters, regardless of ambient temperatures. The pre‐optic area of the hypothalamus is chiefly responsible for this process, integrating afferent thermal information from skin and visceral thermoreceptors along with direct hypothalamic warming/cooling signals to drive vascular, endocrine/metabolic and behavioural responses to thermal challenges [3]. A detailed review of the exact neural mechanisms underlying temperature sensing in the hypothalamus is beyond the scope of this discussion, but is expertly reviewed elsewhere [4], and summarised in Figure 2.

Deviation above or below pre‐defined ‘set points’ (which can temporarily become altered, in the presence of endogenous pyrogens during infection, for example) generates a variety of physiologic and behavioural responses. Hypothermia promotes peripheral vasoconstriction, erector pili muscle contraction, thermogenic shivering, sympathetic excitation to brown fat stores inducing brown fat thermogenesis, hypothalamic–pituitary axis activation to increase thyroxine production and basal metabolic rate and huddled body positioning. Conversely, a rise in core temperature above the set point leads to peripheral vasodilation, sweating and relaxation of the erector pili muscles to aid in heat dissipation.

### 2.2 What Is Hypothermia and How Does It Affect the Body?

Hypothermia is defined as a core temperature of less than 35°C^6^. It can be further divided into mild (32°C–35°C), moderate (28°C–32°C) and severe (< 28°C), with symptoms varying according to severity [5]. Hypothermia drives widespread cellular injury secondary to water crystallisation, membrane disruption, and protein changes. Secondary ischaemic injury due to vasoconstriction, endothelial damage and thrombosis is also common [6]. The magnitude of physiological derangement varies depending on severity. In mild hypothermia, catecholaminergic drive generally promotes tachycardia and hypertension, while moderate to severe hypothermia causes degradation in cardiovascular function, producing progressive bradycardia and hypotension [7]. CNS metabolic rate drops by about 6% for each 1°C temperature drop, producing behavioural change, confusion, ataxia and progressive obtundation [7]. Respiratory drive becomes depressed, producing respiratory acidosis. Electrolyte imbalance also often ensues due to ‘cold diuresis’, reduced renal perfusion impairing renal tubular function and occasionally shivering‐induced rhabdomyolysis. Coagulopathy, secondary to platelet and coagulation factor dysfunction, is also common.

### 2.3 What Causes Hypothermia?

Hypothermia can be broken down broadly into either exogenous (environmental exposure), endogenous (reduced physiologic compensatory mechanisms), or mixed. Certain groups are particularly susceptible either because of deficient compensatory mechanisms, for example, the elderly, impaired perception (alcohol/drug intake) or reduced thermal insulation or endothermic potential (homelessness, reduced body fat, malnutrition). Other endogenous causes include systemic infection and sepsis—especially gram‐negative bacteraemia [8]—hypovolaemic shock, metabolic disturbance such as hypoglycaemia or diabetic ketoacidosis, endocrinopathies such as hypothyroidism, adrenal failure or hypopituitarism and renal or heart failure. A summary of non‐infectious causes of temperature disturbance, alongside clues to aid in their identification, is provided in Table 1.

**TABLE 1:** Non‐infectious causes of hypo‐ and hyperthermia with diagnostic clues in *italics*.

Aetiology		Hypothermia	Hyperthermia
Central Neurological	Structural/vascular	**Diffuse/diencephalic lesions:** ischaemic; haemorrhagic; neoplastic; traumatic. *Central fever: Persistent temperature elevation, without diurnal fluctuations, resistant to antipyretics, in the setting of a known cerebral insult*	
	Inflammatory	**Demyelination:** multiple sclerosis; NMOSD. *Episodic spontaneous hypothermia if disease affecting thermoregulatory pathways (*e.g., *hypothalamus/spinal cord); compatible neuroimaging +/− history/examination features suggestive of other neuro‐inflammatory episodes* **Autoimmune/Paraneoplastic encephalitis** (e.g., LGI1, Ma2, anti‐GAD, anti‐NMDAR, Hashimoto Encephalitis): *Different syndromes according to causative antibody. Symptoms include mental status change, neuro‐psychiatric symptoms, seizure, abnormal movements. Diagnosis: Compatible clinical, neuroimaging, CSF and antibody profiles +/− associated cancer/teratoma*. **Systemic** (e.g., rheumatological/granulomatous/connective tissue disease): *Systemic manifestations (*e.g., *inflammatory arthritis, sicca syndrome), radiological findings (*e.g., *hilar lymphadenopathy), suggestive serological tests*	
	Neurodegenerative	**Parkinson's disease/Lewy body dementia/Multiple system atrophy:** Pre−/postganglionic autonomic disturbance. *Motor Parkinsonism, non‐motor features (anosmia, constipation, orthostatic hypotension, hallucinations, urinary symptoms)*	
	Neuro‐developmental	**Shapiro Syndrome/variants:** Episodic hypothermia, hyperhidrosis, callosal agenesis. *Paroxysmal temperature disturbance. Supportive imaging without evidence of an alternative cause*	
	Other	**Migraine** *Hypothermia documented during typical migraine attacks*	
Peripheral/Autonomic Neurological		**Spinal cord injury:** Impaired afferent sensing and efferent shivering/vasomotor responses; proportional to injury level *Motor/sensory/sphincter symptoms suggestive of spinal cord injury*	
		**Peripheral/autonomic neuropathy** (all causes): impaired sensing or efferent failure. *Relevant history/imaging/serology*	**Anhidrosis syndromes:** CIPA, Ross syndrome, ectodermal dysplasia. *Impaired sweating, severe hyperthermia during exertion/hot environments*
Endocrine		**Endocrinopathy:** Hypothyroidism (myxoedema coma), adrenal failure, hypopituitarism. *Refractory hypotension, bradycardia, skin changes, GI disturbance, electrolyte abnormalities*	**Thyrotoxicosis:** *Tachycardia, tremors, high‐output failure* **Pheochromocytoma:** *Episodic hypertension, diaphoresis*
Metabolic/Systemic		**Metabolic disturbance:** Hypoglycemia, diabetic ketoacidosis, uremia, hepatic encephalopathy **Nutritional:** Wernicke's encephalopathy; severe malnutrition. *Supporting history and imaging; hypovitaminosis*	**Excessive muscle activation:** status epilepticus; prolonged/severe catatonia **Severe dehydration:** profound volume depletion limiting sweating. *Dry skin and mucous membranes, hypotension, hyperosmolar serum*
Toxic/Iatrogenic		**Sedatives/depressants:** Alcohol; opioids; barbiturates; phenothiazines. These impair thermal perception and judgement, and induce vasodilation	**Hypermetabolic/dysautonomic syndromes:** Neuroleptic malignant syndrome (*antipsychotics*); serotonin syndrome (*serotonergic agents*), malignant hyperthermia (*anaesthetics*) **Sympathomimetics:** Amphetamines; cocaine **Anticholinergics:** Inhibited sweating **Oxidative phosphorylation uncouplers:** salicylate toxicity **Drug fever:** Type III drug reaction to antimicrobials, NSAIDs, anti‐neoplastics *Exposure to new medication in last week, pulse‐temperature dissociation + inappropriate sense of wellbeing [drug fever]*
Dermatological		**Loss of skin barrier:** severe burns; exfoliative dermatitis; erythroderma. Excessive heat loss	
Environmental		**Exposure:** cold environments. *Compounded by age, intoxication, reduced body fat, immobility or homelessness*	**Heat stroke:** Classic (impaired loss in vulnerable populations). *Hot, dry skin*. Exertional (extreme heat production in hot environments). *Diaphoresis*
Malignancy^a^			**Lymphoma, Leukaemia, Renal cell carcinoma** *Underlying malignancy; NSAID responsive*

Abbreviations: CIPA, congenital insensitivity to pain with anhidrosis; CSF, cerebrospinal fluid; GAD, glutamic acid decarboxylase; LGI1, leucine‐rich glioma‐inactivated 1; NMDAR, N‐methyl‐D‐aspartate Receptor; NMOSD, neuromyelitis spectrum disorder; NSAID, non‐steroidal anti‐inflammatory drug.

^a^ Malignancies, while causing fever due to release of endogenous pyrogens, generally do not cause true hyperthermia.

### 2.4 Which Neurological Disorders Predispose to Thermoregulatory Dysfunction and When Should Central Thermoregulatory Disorders Be Suspected?

Insults to any part of the thermoregulatory pathway (temperature sensing, response coordination, or effector mechanisms) can impair the response to thermal challenges. Peripheral neuropathies and dorsal root ganglionopathies can impair accurate temperature sensing from the peripheries, but aside from rare genetically driven cases of congenital insensitivity to pain with anhidrosis (CIPA) or Ross syndrome [9], rarely produce clinically overt thermoregulatory abnormalities. Spinal (especially cervical) cord insults not only impair afferent input to hypothalamic thermoregulatory centres but can be associated with efferent thermoregulatory dysfunction due to decreased muscle mass, impaired shivering responses, and dysfunctional peripheral vasomotor responses; the magnitude of thermoregulatory abnormality is generally proportional to the level of the insult, with high cord injuries faring worse. Immune mediated, neurodegenerative, or focal lesions of brainstem or hypothalamic regions may directly affect the ability of the hypothalamic thermostat to operate appropriately.

Clinicians should suspect a central thermoregulatory disorder when a patient presents with unexplained hypothermia or hyperthermia, particularly in the absence of environmental triggers or identifiable infection, endocrinopathy, or systemic organ malfunction. As such, it is largely a diagnosis of exclusion. Additional neurological signs (such as other autonomic dysfunction or altered mental status) may point to a central cause but can also be a direct result of thermal derangements. Structural brain imaging in such cases is paramount to search for potentially causative hypothalamic or brainstem anomalies.

### 2.5 What Is Shapiro Syndrome and How Is It Managed?

Periodic hypothermia syndromes are uncommon and generally point towards diencephalic dysfunction. Centrally driven episodic hypothermia has been reported in a range of conditions including migraine [10], HIV [11], Parkinson disease [12] and multiple sclerosis [13]. A rare but important consideration should also be Shapiro syndrome, a disorder characterised by recurrent, spontaneous, periodic hypothermia and hyperhidrosis in conjunction with agenesis of the corpus callosum [14].

Shapiro syndrome was first described in 1969 [14] in two patients who presented with spontaneous episodic hypothermia and hyperhidrosis with radiographic evidence of agenesis of the corpus callosum. Since the initial description, the phenotype has expanded to include other autonomic disturbances such as hypertension or hypotension, altered consciousness, and metabolic derangement [15, 16]. Furthermore, of the approximately 60 cases of Shapiro syndrome described in the literature, 50% had no evidence of callosal dysgenesis [15]—these are considered a variant of the syndrome, sometimes referred to as episodic spontaneous hypothermia, particularly affecting older patients [17].

The pathophysiologic basis of Shapiro syndrome remains uncertain, though dysgenesis of the corpus callosum alone is insufficient. Hypothalamic involvement in Shapiro syndrome is supported by findings of hypothalamic neuronal loss in some cases [18], although often there is no abnormality on structural imaging. Shapiro's hypothesis of diencephalic epilepsy does not seem to have borne out: patients very rarely have any abnormality on EEG and generally do not respond to treatment with anticonvulsants [19]. Neurotransmitter dysfunction [20] and hypermelatonaemia [21] have been proposed as potential pathomechanisms, but the cause‐effect relationship of these is difficult to disentangle. In support of the former is the occasional response to prophylactic neurotransmitter‐modulating treatments, including clonidine, clomipramine and cyproheptadine [19]. Our patient commenced treatment briefly with clonidine, but this was not continued due to poor response, issues with non‐compliance and episodes of hypotension.

While virtually any neurological disease affecting cognition, sensation or movement may make an individual more susceptible to thermal dysregulation, as this case highlights, periodic hypo‐ and hyperthermia should prompt consideration of a central disorder once infection and systemic disease have been excluded. Diagnostic accuracy is important as management strategies (both pharmacological and non‐pharmacological) may vary according to the identified cause.

## Author Contributions

N.K. was involved in conception and design of this manuscript, data collection and drafting the original version of the manuscript. J.J. and S.W. were involved in analysis and interpretation of the data, and critical revision of the manuscript for intellectual content. E.M. was involved in conception and design of this manuscript, data collection and critical revision of the manuscript for intellectual content.

## Funding

The authors have nothing to report.

## Conflicts of Interest

The authors declare no conflicts of interest.

## Supporting information


**Table S1:** Summary of basic blood workup at time of presentation.
**Table S2:** Summary of additional laboratory investigations.

## Data Availability

Data sharing not applicable to this article as no datasets were generated or analysed during the current study.
