# Supplementary material for: Recurrent Hypothermia and Autonomic Dysfunction Secondary to Shapiro Syndrome
Source: Ann Clin Transl Neurol. 2026 Apr 29:10.1002/acn3.70419. Online ahead of print. doi: 10.1002/acn3.70419 (PMC13394928; doi:10.1002/acn3.70419)
Supplement: Supplementary file 1 — Table S1: Summary of basic blood workup at time of presentation. Table S2: Summary of additional laboratory investigations. [file ACN3-9999-0-s001.docx]

**Supplementary Table 1: Summary of basic blood workup at time of presentation**

|  | **Value** | **Reference range** |
| --- | --- | --- |
| Haemoglobin | 132 | 130–165 g/L |
| White cell Count | 4.99 | 4–11 x 10^9^/L |
| Platelets | 362 | 150–450 x 10^9^/L |
| Sodium | 126 | 135–145 mmol/L |
| Potassium | 3.0 | 3.5–5.3 mmol/L |
| Urea | 4.3 | 2.5–7.8 mmol/L |
| Creatinine | 99 | 61–123micromol/L |
| AST | 34 | 10–50 IU/L |
| ALT | 18 | 5–55 IU/L |
| ALP | 89 | 30–130 IU/L |
| Bilirubin | 10 | <20 micromol/L |
| Adjusted Calcium | 2.28 | 2.15–2.6 mmol/L |
| Magnesium | 0.80 | 0.7–1.0 mmol/L |
| Phosphate | 0.9 | 0.8–1.4 mmol/L |
| Albumin | 47 | 35–50 g/L |
| CRP | <1 | <5 mg/L |
| TSH | 0.53 | 0.27–4.2 mIU/L |
| Prothrombin time | 10.1 | 10–12 sec |
| APTT | 27.4 | 20–29 sec |

**Supplementary Table 2: Summary of additional laboratory investigations**

|  | **Value** | **Reference range** |
| --- | --- | --- |
| **Blood** | | |
| Infectious serology (HIV, Hepatitis, Syphilis, Trypanosoma, Borrelia Burgdorferi, Schistosoma, Whipple’s | Negative |  |
| Strongyloides serology | Positive |  |
| Autoimmune serology (ANCA, ANA, ENA, anti-dsDNA, IgG subclasses) | Negative |  |
| Paraneoplastic antibodies (Hu, Ri, Yo) | Negative |  |
| Autoimmune encephalitis panel (AMPA1, AMPA2, GABAR; VGKC, NMDAR, CASPR2, CV2/CRMP, IA2, PNMA2/Ta, amphiphysin, DPPX, IGLON5) | Negative |  |
| Early morning Cortisol | 404 | 133–537 nmol.L |
| Plasma ACTH | 13 | 0–46 ng/L |
| Plasma Metadrenaline | 446 | 80–510 pmol/L |
| Plasma normetadrenaline | 1020 | 120–1180 pmol/L |
| Plasma 3-methoxytyramine | <120 | <120 pmol/L |
| Plasma osmolality | 258 | 280–295 mmol/L |
| **Urine** | | |
| Osmolality | 113 | 50–1200 mOsm/kg |
| Sodium | 13 | >20 mmol/L |
| 24h normetadrenaline | 1.85 | <3.7 micromol/24h |
| 24h metadrenaline | 0.47 | <1.3 micromol/24h |
| 3-methoxytyramine | 1.14 | <2.6 micromol/24h |
| **CSF** | | |
| WCC | <5 | <5 |
| RCC | <5 | <5 |
| Glucose | 3.5mmol/L |  |
| Protein | 0.60 | 0.25–0.45 g/L |
| Oligoclonal bands | Negative |  |
| Orexin | >110 but <200 | >200 pg/mL |
| Viral PCR (EBV, CMV, VZV, HSV, enterovirus) | Negative |  |
